# Developing a Quality Benchmark for Determining the Credibility of Web Health Information- a Protocol of a Gold Standard Approach

**DOI:** 10.3389/fdgth.2021.801204

**Published:** 2021-12-23

**Authors:** Lubna Daraz, Sheila Bouseh

**Affiliations:** School of Library and Information Science, Faculty of Arts and Sciences, University of Montreal, Montreal, QC, Canada

**Keywords:** online, credibility, assessment, health information, benchmark, protocol

## Abstract

**Background:** The current pandemic of COVID-19 has changed the way health information is distributed through online platforms. These platforms have played a significant role in informing patients and the public with knowledge that has changed the virtual world forever. Simultaneously, there are growing concerns that much of the information is not credible, impacting patient health outcomes, causing human lives, and tremendous resource waste. With the increasing use of online platforms, patients/the public require new learning models and sharing medical knowledge. They need to be empowered with strategies to navigate disinformation on online platforms.

**Methods and Design:** To meet the urgent need to combat health “misinformation,” the research team proposes a structured approach to develop a quality benchmark, an evidence-based tool that identifies and addresses the determinants of online health information reliability. The specific methods to develop the intervention are the following: (1) systematic reviews: two comprehensive systematic reviews to understand the current state of the quality of online health information and to identify research gaps, (2) content analysis: develop a conceptual framework based on established and complementary knowledge translation approaches for analyzing the existing quality assessment tools and draft a unique set of quality of domains, (3) focus groups: multiple focus groups with diverse patients/the public and health information providers to test the acceptability and usability of the quality domains, (4) development and evaluation: a unique set of determinants of reliability will be finalized along with a preferred scoring classification. These items will be used to develop and validate a quality benchmark to assess the quality of online health information.

**Expected Outcomes:** This multi-phase project informed by theory will lead to new knowledge that is intended to inform the development of a patient-friendly quality benchmark. This benchmark will inform best practices and policies in disseminating reliable web health information, thus reducing disparities in access to health knowledge and combat misinformation online. In addition, we envision the final product can be used as a gold standard for developing similar interventions for specific groups of patients or populations.

## Introduction

The current pandemic of COVID-19 has changed how health information (HI) is distributed through online platforms. These platforms, including social media, have played a significant role in providing patients and the public with a vast amount of questionable information. This was evident in a recent rapid systematic review completed by our research team to explore the current state of the evidence of HI relevant to COVID-19. This study revealed that social media (70% of studies) had played an integral role in conveying information to the patients/public (PAP). Simultaneously, there are growing concerns that much of the information is not credible, impacting patient health outcomes, costing human lives, and causing tremendous resource waste (1–3). With the increasing use of online platforms, PAP requires a new way of filtering “credible” from “questionable” HI and needs to be empowered by strategies to navigate online disinformation.

A study by the Reuters Institute surveyed six countries and reported that one-third of the online users saw false or misleading information about COVID-19 (2, 4). The authors also noted that people with a low health literacy level are more likely to rely on the web than other information sources. Therefore, the more PAPs go on the web to meet their HI needs, the more unreliable information can be provided by deceitful HI producers. This vulnerable situation of PAP has created urgent research needs to ensure efficient access to reliable web-based content.

Approximately 35% of patients who seek medical information on the web do not visit a physician to verify the information's accuracy (5). Billion dollars are wasted on unproven therapies and deceptive cures that cause a delay in the receipt of evidence-based treatments. In addition to the poor readability, many websites have non-evidence-based and biased information due to the writers' or their sponsors' financial and intellectual conflict of interest. While online sources can contribute to “credible” information and combat “misinformation”; there remain questions about the contents and source's reliability. Therefore, the current work is timely and requires immediate attention from the scientific community.

The crucial state of the lack of reliability of online HI has not improved; instead declined rapidly in the last decade (1, 6). To address this issue, organizations and individuals have developed numerous quality assessment tools, such as DISCERN (7), the HON Code of Conduct (8), the Journal of the American Medical Association (JAMA) benchmark (9) and so on. A review conducted in 2009 also identified a list of quality assessment tools, and the numbers of these tools are increasing gradually (10). Another research team who reviewed the literature for existing tools concluded a need for a tool for assessing online HI specific to optimal aging (11). This tool included thirteen questions with predominantly scientific terminologies challenging for a lay PAP. For example, one of the questions is, “Is the Web resource informed by published systematic reviews/meta-analyses?” (11). Similarly, DISCERN, which is widely used by the scientific community, was developed for a specific context and contained numerous questions which can be challenging to use for an individual with low literacy to apply (11). Additionally, there is no evidence of the degree of the usefulness of these tools and how PAP is utilizing them. Many of these tools include numerous criteria, are not user-friendly for PAP with low health literacy, and more importantly, have not been validated by PAP extensively (10, 12). At the same time, the reliability and validity of these tools are often not evident (10, 13). Our study will address this gap. Since the existing tools were developed based on the developers' specific needs and, maximum did not target PAP, our proposed intervention will be designed to assess HI relevant to the content, source, ease of use, and readability for PAP. The quality benchmark developed from this project will comply with the recommendations of AMA and NLM that the reading level of HI disseminated to PAP should be Grade 6 or below (14, 15).

Each of the existing tools consists of quality measures or criteria such as “authorship,” “accessibility,” “usability,” and so on to determine the level of reliability of HI. These criteria are presented with questions representing the accompanying theme or scoring classifications to rate the information. We define these criteria as quality “domains” or “determinants of reliability.”

The existing tools lack the information regarding how the quality criteria were developed, selected, and validated and often did not assess the website's actual content. As a result, the websites may score high as measured by the domains, while content quality may remain low (16). More importantly, one of the significant criteria, “readability,” vital to PAP with a low literacy level, is often not measured by those tools. This significant gap will be addressed in the quality domains that we intend to develop.

To our knowledge, no one has used a systematic approach to develop an evidence-based quality assessment tool that is patient-centered, easy to use, and engages relevant stakeholders. It is also unclear if theories and frameworks from HI seeking and knowledge translation (KT) have informed the development of the existing tools. As a result, concerns regarding the underlying concepts used in developing the tools and a lack of agreed determinants of reliability persists. This uncertainty suggests the need for an in-depth analysis of the existing tools for relevance, readability, and usability and developing a new tool that can be used as a gold standard.

## Methods and Analysis

**Figure 1:**
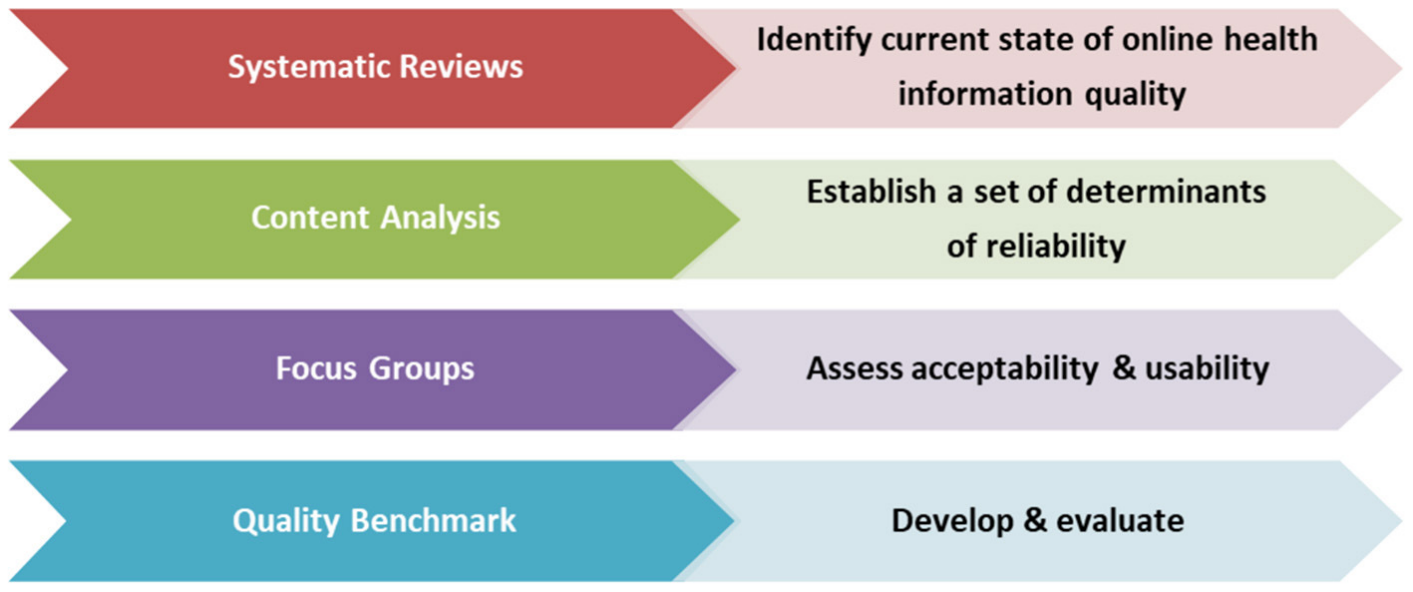
Methods to develop quality benchmarks for use by patients and public.

To assure the rigorous development of the evidence-based patient-centered quality benchmark, we have planned a multi-step research endeavor using four separate studies. Figure 1 below shows the phases of the project with the purpose of each study.

### Timeline

We anticipate that this project will be completed in three years. The two systematic reviews were completed in one year. Content analysis will take six months, focus groups will take one year, and development and validation of the benchmark will take six months. Additional time will be needed for the dissemination and implementation of the intervention.

### Theoretical Underpinnings of the Project

The theoretical perspective that informed this multi-stage project is the association between HI seeking behavior and factors correlated with health outcomes, such as reliability and readability of web-based HI. One of the well-known models proposed by Johnson, the “Comprehensive Model of Information Seeking (CMIS),” was developed in the context of HI seeking (17). The model emphasizes the information carrier factors, which are the characteristics and usefulness of a particular source that influence an individual's decision to seek information from that source. In considering the attributes of carriers, Johnson (1995) refers to the user's perception of their credibility and authority and the accuracy and comprehensibility of the information (17). The stages of this proposal are thus informed by this model to uncover the information carrier (the web) factors consistent with the patient's needs to develop the intervention successfully.

### Study 1- Systematic Reviews

The study team has completed ***two*** comprehensive systematic reviews to understand the current state of the quality of online HI targeting PAP and to identify research gaps (18, 19). The studies established that an individual requires a Grade 12 or college education to understand online HI. This finding means that most websites that provide HI, are not understood by a large number of PAP. We also found suboptimal quality (44%) across websites, suggesting a significant gap in evidence-based online HI provided to PAP (31). The findings from the reviews have informed the design of the following methods to develop the intervention.

### Study 2- Content Analysis

#### Rationale

The variation in quality according to different quality tools justifies content analysis. Some tools share similar quality domains, but others do not. Therefore, it is essential to investigate how the domains were defined, selected, consistent, and informed by theory. Identifying the necessary and unique domains can better explain the determinants of reliability.

#### Method

Currently, we are conducting a content analysis of selected quality assessment tools. We have consulted several established and complementary approaches to develop a conceptual framework (Table 1) for analyzing the selected tools' content (definition, scoring criteria, consistency, readability) (13, 16, 20). The Delphi method, a structured, rigorous, and credible technique with a systematic method of obtaining consensus from a panel of stakeholders, is used for the content analysis (21). Our study panel consists of five team members, including the lead investigator (LD), clinicians, knowledge translation (KT) researcher, and 30 PAP. The KT researcher is the facilitator, and six rounds of iterative processes have been used. Table 2 describes the steps of each round for selecting the final list of quality domains for the benchmark. Similar steps will be used to generate different groups of scoring criteria.

**Table 1:** Conceptual framework for analyzing contents.

**Steps**	**Descriptions**
Step 1	Identify a list of domains in each tool
Step 2	Group the domains that overlap across tools in specific categories to reflect core themes. For example, domains with similar definitions or sub-themes should be categorized under a particular theme, such as Accuracy, which may be defined as a sub-theme like accurate or reliable.
Step 3	Identify domains that are unique or lack specificity (i.e., do not overlap across tools).
Step 4	Systematically evaluate psychometric properties or validity testing when provided.
Step 5	Explore heterogeneity (inconsistency across tools) in terms of population, domain type, number of items, scoring criteria, and format.
Step 6	Present convergence of themes into a final list of unique and parsimonious domains.
Step 7	Determine the scoring criteria with intuitive and precise interpretation.

**Table 2:** Guideline for delphi method.

**Steps**	**Descriptions**
Round 0	A minimum of 2 research team members will generate a list of domains and accompanying questions from the selected quality assessment tools for the subsequent steps.
Round 1	A minimum of 2 team members (presumably the lead researcher and a KT expert) will analyze the contents of each tool and compare them across the tools. A list of overlapping domains will be classified as “common” domains. A similar method should be used to identify domains that lack specificity, classified as “unique” domains. Disagreement will be resolved after discussion.
Round 2	A minimum of 3 independent reviewers (presumably the lead researcher, a clinician, and a KT expert) will evaluate the set of “common and “unique” domains along with questions. The facilitator will compose their input in terms of the reviewers” perspectives and recommendations based on their understanding of the health information needs of the stakeholders.
Round 3	An online group discussion will be conducted where the facilitator will present the independent reviews to the panel members. The members will have an opportunity to compare their responses with other independent reviewers' responses. Following an active discussion, the facilitator will draft a list of domains and scoring criteria to present to the experts and patients and public.
Round 4	A group of experts will be comprised of clinicians, health information providers, KT experts, and the lead researcher who will be presented with the domains and scoring criteria from round 3. They will review the list of the domains along with questions and scoring criteria for usability and relevance to the stakeholders. Finally, the experts will be asked to approve or modify the existing list to be used in round 6. This round will be conducted online.
Round 5	Different groups of patients and public will meet online/in-person in their sub-category (i.e., age, race, gender, health condition etc.) focus group where the facilitator will present the domains along with descriptions, questions and scoring criteria. Then, the participants will discuss their understanding of the domains and decide the final list based on their health information needs and preferences. Finally, the facilitator will produce a report on participants' responses, with their preferences for the final round.
Round 6	The research team members led by the primary investigator will review both the responses and recommendations of the experts and patients and public. A final list of domains and scoring criteria will be established in this step. Then, a representative group of patients and public will validate the final list using the “Member checking” technique.
Deliverable	A final list of quality domains and scoring criteria for the quality benchmark.

#### Results

After completing the content analysis of the existing tools, the team will draft a unique list of domains (determinants of reliability) used to conduct focus groups and develop the benchmark.

### Study 3- Focus Groups

#### Rationale

The majority of the quality assessment tools were developed for a specific audience such as healthcare providers, professionals, site managers and without direct involvement by PAP (11, 12). Patients/public preferences and values were not reflected in validating the quality criteria (22). For the qualitative study, we will engage relevant stakeholders to test the acceptability and usability of the quality domains and scoring criteria. During the evaluation, the participants will reflect their preferences into a standard patient user-friendly benchmark. Since PAP is our primary target population, utilizing a focus group approach will be more feasible and beneficial. It may lead to interactions between individuals that provide additional insight and a deeper understanding of the phenomena being studied (23, 24). We will use individual semi-structured interviews for the HI providers to receive additional feedback on the intervention development.

#### Method

Focus Groups with patients/public.

#### Setting

Although the primary study location is in Montreal, Quebec, we will recruit a representative sample of PAP across Canada through the gatekeepers of patients/public.

#### Sampling

Purposeful criterion sampling will be used to select participants for their potential to be most informative about the phenomenon under study (23, 24). For the focus group study, it is essential to select participants who have access to the Internet and experience searching for HI online. They will provide relevant and sufficient information about their preference for quality domains. The participants' inclusion criteria are: men or women, 19 or older, have lived with an illness or provide support to someone with an illness, have experience searching for HI online, and communicate in English or French. We have selected this group of PAP based on our study target population and the study's objective. The exclusion criteria are: do not have experience searching for online HI or do not have access to computers and clinicians or healthcare professionals. According to our objective, we are developing the benchmark for PAP. Therefore, healthcare professionals cannot be among the target population. We will attempt to recruit participants with different socioeconomic backgrounds (gender, race, age, social class, income, education, employment status, health condition) to reflect diverse persons' experiences with online HI, thus achieving health equity. For this study, we have chosen a sample size of 30 to reach data saturation (23, 24). There will be two language groups (1 in Quebec with 15 participants, 1 across Canada with 15 participants) with PAP. We will conduct a series of smaller focus groups (5 sessions) with different categories of people (6 participants in each) to overcome the challenge of decreasing the sensitivity of identifying trends of sub-categories.

#### Recruitment Strategies

Participants will be identified through gatekeepers for patients, caregivers, and the public in Quebec Province and Canada. As an affiliated member of the university, the PI has access to the patients' population from the university-affiliated teaching hospitals for this project. Also, the PI will use her network affiliated with organizations, patient engagement units, advocacy groups, public health education centers, community support groups, public libraries etc., for additional recruitments from Quebec and across Canada. We will also use a snowball sampling strategy to reach participants with characteristics underrepresented in the group (23–25). For example, women search more online HI than men (26). Therefore, we will recruit a representative sample of women to receive their feedback for the benchmark. From general PAP (new immigrants, women, vulnerable etc.), we have received a commitment for potential participation in this study. Their participation will ensure health equity in this study.

#### Data Collection

The focus groups will be a 2-h workshop with web conference capability for participants who may join remotely based on the pandemic status. There will be five focus groups with each sub-category of people. Once the participants agree to participate in the study, the research associate (RA) will contact them to prepare for the workshop and answer any preliminary questions. The workshop will include a brief didactic to allow interactive discussions with breaks. The didactic will cover the following topics (1) an introduction to online HI, (2) advantages and disadvantages of online HI, (3) the current state of online HI, (4) a brief introduction to quality tools and domains, and (5) selected quality domains from content analysis –explanation, definitions, and scoring criteria. Doctoral students and the RA will deliver the workshops. Throughout the presentations, participants will be encouraged to engage in the discussions. Following the presentation, participants will be asked open-ended questions. The analysis of Study 2 will help us develop questions for focus groups regarding their experience with English and French online HI, content, format, and quality. We will also ask them whether the domains are feasible, understandable, user-friendly, and easy to elicit by a layperson. Examples of questions are provided in Table S1 (Supplementary Document), which will be pilot tested and further developed. With the participants' permission, we will use an online/tape recorder to ensure accuracy in capturing information during the focus groups. Before the focus groups, a questionnaire will be sent to participants to collect demographic data (age, race, gender, employment status, marital status, ethnicity, income, and education).

### Semi-structured Interviews With Health Information Providers

#### Setting, Sampling and Recruitment

We will recruit representatives from organizations that produce HI for PAP. We chose the following organizations/sources because they appear in the top 10 hits in Google when a health condition or treatment is searched: (1) Mayo clinic.com, (2) WebMD, (3) Santé Public, (4) MedlinePlus, (5) Health Canada, (6) Wikipedia, (7) Healthline.com, (8) KidsHealth.org, (9) camh.ca, and (10) Heart and Stroke Foundation of Canada. The study team will invite several representatives from each of these organizations responsible for producing HI for the PAP, which will ensure the final recruitment of 10 participants.

#### Data Collection

The interviews will be conducted individually during a 1-h phone/zoom conference call. The interview materials will be sent one week in advance to participants (including the proposed quality domains). The interview questions will be open-ended and about acceptability and feasibility. Examples of questions are provided in Table S1 (Supplementary Document), which we will further develop. Health information providers' interviews will also inform the development of the quality benchmark customized to PAP. Doctoral students and RA will facilitate the interviews with the participants.

#### Data Analysis

The analysis will be conducted concurrently with the collection of the interview data. We will use the constant comparison analysis for this proposal, appropriate for multiple focus groups within the same study (27). This analysis comprises three stages: (1) data are chunked into small units where researchers add a descriptor or code to each of the units, (2) codes are grouped into categories, and (3) develop one or more themes that express the content of each of the groups. This technique allows assessment of the themes that emerged from one group and those that emerged from other groups and ensures data saturation or theoretical saturation. For example, since women search for HI more than men, we will perform a subgroup analysis based on gender. We will also perform subgroup analysis for PAP with low health literacy and the level of education. A qualitative data synthesis software, NVivo 12 Pro will be used for data analysis.

At the end of this stage, the content of the intervention will be determined that is validated by the stakeholders. An ethics approval will be obtained from the University of Montreal Institutional Review Board (IRB) to recruit participants.

### Study 4- Develop and Evaluate the Quality Benchmark

Based on the stakeholders' (PAP and HI providers') acceptability and validity from conducting the focus groups, a unique set of determinants of reliability (Quality Benchmark) will be finalized, along with a preferred scoring classification. A graphic designer with expertise in designing patient materials for laypersons will generate the benchmark content (domains and scoring criteria) appropriate for our target population. Finally, we will develop both an electronic and a printed version of the benchmark.

#### Readability Assessment of the Quality Benchmark

We will evaluate the reading level (the ease of understanding written text) of the benchmark's content using “The Flesch Reading Ease (RE)” score maps (28). The readability should receive Grade 6 or below.

#### Member Checking

We will use the “Member checking” technique to validate participants' views of the credibility of the domains/scoring criteria and interpretations of their feedback into the benchmark (23). A representative sample of 10 participants from each language group will be contacted for this validation. This strategy will help improve the focus groups' accuracy, credibility, validity, and transferability (39).

#### Usability Testing

A usability test will be conducted with a representative sample (6 from each PAP group). Each participant will receive three documents: (1) two websites, (2) the draft quality benchmark, and (3) a feedback form (Supplementary Document). Participants will use the benchmark to evaluate the websites and provide feedback after the completion of the evaluation. The RA will provide guidelines and monitor this process for accuracy and answer any questions the participants may have during the completion of the evaluation task. We will incorporate the feedback from the participants into account for the final revisions of the benchmark. The graphic designer will draft the final version of the benchmark in consultation with the investigators.

The outcome will be a quality assessment benchmark that will assist PAP in evaluating and accessing credible health information online.

### Study Team

The team is ideally situated to conduct the proposed project. The project will be completed at the University of Montreal, School of Library and Information Science. The university has an extensive network of affiliated hospital centers that support fundamental, clinical, applied, evaluative, or interdisciplinary research. The lead of this project has 14 years of experience in knowledge synthesis, KT, and methodology expertise in assessing web health information, focus groups, mixed methods, tools, guidelines, and patient training curriculum development. The investigating team consists of clinical epidemiologists, clinicians, Canada Research Chair, data science and methodology experts, interdisciplinary scholars with expertise in a vulnerable population, and knowledge translation experts.

### Potential Challenges and Mitigation Strategy

We expect several challenges for conducting the studies for which we have adopted a mitigation strategy. In the focus groups recruitment, we anticipate a lack of representation of diverse groups. Typically, a more diverse focus group decreases the sensitivity to identify any trends of sub-categories. Therefore, the team will adopt multiple strategies to overcome this challenge. For example, a series of smaller focus groups (5–8) with different categories of participants is recommended to satisfy the sensitivity to detect a trend. Our study team has decided on five focus groups with six participants in each sub-category. In the situation where the participants have limited experience with the topic, we will conduct a focus group with a larger number of participants (minimum of 10) for that specific category. Another challenge we expect is a lack of commitment among HI providers. So, we will invite several representatives from 10 different organizations to ensure sufficient participation.

## Dissemination

The research team has proposed to actively engage stakeholders in the study. For example, workshops with the PAP will encourage active participation and reflect their values and preferences into the benchmark. This strategy will also improve awareness, thus increasing the PAP's knowledge regarding the reliability of online HI. In addition, we will invite HI providers to participate in the focus group interviews to promote engagement and contribute to their experiences. We will adopt a list of knowledge transfer strategies to increase the accessibility and flow of the knowledge gained from the study—for example, peer-reviewed journal publications, conference presentations (i.e., Technology, Knowledge & Society conference), professional associations (i.e., Association for Information Science and Technology), Twitter, Linked In, and Facebook posts to reach a wider audience. A pdf version of the benchmark will be distributed to HI providers, librarians, and patient advocacy groups (i.e., Canadian Medical Association's Patient Advocacy Community, the Canadian Pain Society) that may distribute it freely to their members through publication or reference to its availability. The investigators will also deliver workshops among the collaborators [i.e., Mayo Clinic, Centre de recherche en santé publique (CreSP), International Observatory on the Societal Impacts of AI and Digital Technology (OBVIA)]. The goal is to increase awareness to exceptionally diverse PAP and HI providers who will benefit from the research, thus increasing the intervention's uptake. These activities are designed based on the Integrated Knowledge Translation (iKT) approach (29).

## Expected Outcomes

This proposed multi-phase project informed by theory will lead to new knowledge intended to inform the development of a patient-friendly quality benchmark. In addition, this benchmark will inform best practices and policies in disseminating reliable web HI, thus reducing access disparities and combating misinformation online. We envision the final product as a gold standard for evaluating online HI used by PAP.

The proposed research activities will also ensure the following outcomes:

- develop and deploy new knowledge to understand health information seeking on the web,
- actively engage diverse persons to create and implement evidence-based solutions to facilitate health information seeking on the web, and
- a first attempt to develop theory-based and validated patient-friendly online health information seeking interventions.

By providing a sampler methodology for developing a patient-centered tool, this work will contribute to future innovations in health information science, public health, and other professions due to their work and their involvement in the healthcare of persons with different health conditions.

This research protocol can also be used as a gold standard to study the credibility of specific health conditions online. In addition, the protocol can be used in non-digital or non-health-related tools development in multidisciplinary research.

## Author Contributions

LD led the development of the protocol and wrote the manuscript. SB contributed to the protocol refinement and editing of the manuscript. All authors contributed to the article and approved the submitted version.

## Funding

This project is funded by a Knowledge Development Grant from the Social Sciences and Humanities Research Council (SSHRC) to LD Grant # RNH01593.

## Conflict of Interest

The authors declare that the research was conducted in the absence of any commercial or financial relationships that could be construed as a potential conflict of interest.

## Publisher's Note

All claims expressed in this article are solely those of the authors and do not necessarily represent those of their affiliated organizations, or those of the publisher, the editors and the reviewers. Any product that may be evaluated in this article, or claim that may be made by its manufacturer, is not guaranteed or endorsed by the publisher.
