# Supplementary material for: Developing a Quality Benchmark for Determining the Credibility of Web Health Information- a Protocol of a Gold Standard Approach
Source: Front Digit Health. 2021 Dec 23;3:801204. doi: 10.3389/fdgth.2021.801204 (PMC8732749; doi:10.3389/fdgth.2021.801204)
Supplement: Supplementary file 1 [file Data_Sheet_1.docx]

**Table 1. Sample Focus Groups and Semi-Structure Interview Questionnaire**

|  |  |
| --- | --- |
| Interview sample questions-Patients/Public | Interview sample questions-Providers |
| - As a user of online health information, please tell us why you use online sources instead of other sources. - Can you please tell us, how do you find health information online? - What kind of information do you look for online (general information about a condition, treatment, symptoms, genetics, alternative treatments, costs, services etc.)? - Do you find online health information useful? If so, in what ways? If not, what is wrong with the information? - Do you know how to determine what is correct and what is wrong health information? How do you know this? - Do you know of any guideline that will help you evaluate the health information available online? - We have presented you with a list of criteria that may help you determine online health information quality.   Based on the discussions during the workshop and your prior experience searching for health information online:   - can you tell us which criteria are easy to understand? - which are easy for you to use? - are there others that you think are important and have been missed? - How is it best to frame these items in the form of a question, multiple choice answers, or a checklist? - Can you think of any barriers to use these quality criteria? For example, language, format, yes/no options. - This phase of the project will help us develop and disseminate a user guide that can be used by users like you to evaluate the quality of online health information.   Do you have any preferences of how the guide should be designed and distributed to potential users? Can you think of any barriers to use the guide? | - Is it possible for you to develop material consistent with the domains we identified from patient/public focus groups? - Is it easy to develop? What are the potential barriers? - Would it be more costly? - Do you have to hire more employees or employees with different skill sets to produce this material, or can you do it with your current staffing? - Do you have to change your literature search and content expert consultations to produce this material? - What would the new approach to generating medical/health information look like? - Any technology-related barriers or facilitators that you anticipate when producing online health information congruent with these quality domains? - This project phase will help us develop a quality benchmark followed by a user guide for patients and public.   Do you have any recommendations of how the benchmark/guide should be designed and disseminated to use for your patient/user population? |

**Quality Benchmark-Usability Test-Feedback Form–Patients/Public**

Usability Test Feedback Form

Evaluating the Quality of Web Health Information

Patients/Public

Please use the Quality Benchmark to evaluate two health information websites. After the completion, please answer the following questions:

1. How useful do you think the Quality Benchmark is?

1 2 3 4 5 6 7 8 9 10

Not Extremely

Useful Useful

1. How easy was to use the Quality Benchmark to help you evaluate the quality of online health information?

1 2 3 4 5 6 7 8 9 10

Not Extremely

Easy Easy

1. Would you use this Quality Benchmark when searching for health information from the web? Please explain.
2. Is there anything in the Benchmark that you would like to change and why? Please explain.
3. Please provide any additional feedback/comments you have.
